# Automatic Extraction and Detection of Characteristic Movement Patterns in Children with ADHD Based on a Convolutional Neural Network (CNN) and Acceleration Images

**DOI:** 10.3390/s18113924

**Published:** 2018-11-14

**Authors:** Mario Muñoz-Organero, Lauren Powell, Ben Heller, Val Harpin, Jack Parker

**Affiliations:** 1 Telematics Engineering Department, Universidad Carlos III de Madrid, Av. Universidad, 30, 28911 Leganes, Spain; 2 School of Health and Related Research, University of Sheffield, Regent Court, 30, Sheffield S1 4DA, UK; l.a.powell@sheffield.ac.uk (L.P.); jack.parker@sheffield.ac.uk (J.P.); 3 Centre for Sports Engineering Research, Sheffield Hallam University, Sheffield S10 2LW, UK; b.heller@shu.ac.uk; 4 Ryegate Children’s Centre, Sheffield S10 5GA, UK; Val.Harpin@sch.nhs.uk

**Keywords:** ADHD, tri-axial accelerometers, deep learning, convolutional neural networks (CNN)

## Abstract

Attention deficit and hyperactivity disorder (ADHD) is a neurodevelopmental disorder, which is characterized by inattention, hyperactivity and impulsive behaviors. In particular, children have difficulty keeping still exhibiting increased fine and gross motor activity. This paper focuses on analyzing the data obtained from two tri-axial accelerometers (one on the wrist of the dominant arm and the other on the ankle of the dominant leg) worn during school hours by a group of 22 children (11 children with ADHD and 11 paired controls). Five of the 11 ADHD diagnosed children were not on medication during the study. The children were not explicitly instructed to perform any particular activity but followed a normal session at school alternating classes of little or moderate physical activity with intermediate breaks of more prominent physical activity. The tri-axial acceleration signals were converted into 2D acceleration images and a Convolutional Neural Network (CNN) was trained to recognize the differences between non-medicated ADHD children and their paired controls. The results show that there were statistically significant differences in the way the two groups moved for the wrist accelerometer (*t*-test *p*-value <0.05). For the ankle accelerometer statistical significance was only achieved between data from the non-medicated children in the experimental group and the control group. Using a Convolutional Neural Network (CNN) to automatically extract embedded acceleration patterns and provide an objective measure to help in the diagnosis of ADHD, an accuracy of 0.875 for the wrist sensor and an accuracy of 0.9375 for the ankle sensor was achieved.

## 1. Introduction

Attention deficit and hyperactivity disorder (ADHD) is a neurodevelopmental disorder, which affects 3 to 5% of school-aged children [1,2]. Children and adolescents with ADHD typically present with symptoms of inattention, impulsivity and hyperactivity, which can have a profound impact on intellectual and social functioning and emotional well-being [3]. The inattention and/or hyperactivity-impulsivity symptoms [4] can have a direct impact on the person’s movements and reactions.

A diagnosis of ADHD (for the International Statistical Classification of Diseases and Related Health Problems (ICD 10)) requires individuals to demonstrate, in more than one setting, a minimum number of symptoms in all three dimensions (inattention, hyperactivity, and impulsivity [5]). The Diagnostic and Statistical Manual of Mental Disorders (DSM-IV [6]) and more recent DSM-5 [4] define only two dimensions (with hyperactivity and impulsivity symptoms included in the same dimension [7]). Current service provision for individuals with ADHD involves a complex assessment and treatment process, requiring clinicians, parents, and education providers to complete multiple questionnaires and interviews detailing their subjective account of symptoms [7]. In recent years, however, there have been attempts to extract measures that correlate with ADHD symptoms from body worn physiological sensors. Some of these objective tools are based on continuous performance tests (CPTs) [8] and electroencephalogram (EEG) [9]. EEG and CPT testing can be costly to administer, requiring expensive equipment and specialized personnel, especially in the case of EEG [10]. Therefore, recently, new objective measures based on inertial sensors such as accelerometers and gyroscopes have been studied [10,11,12]. These previous studies have focused on finding statistically significant differences in the data gathered from ADHD patients and typically developing non-ADHD controls when performing specific and predefined activities.

Although the direct observation of ADHD individuals in controlled environments may be a useful adjunct in diagnosing ADHD, unfamiliar settings may provoke differences in behavior, especially for children, consequently, unobtrusive monitoring of ADHD children in their natural environment while attending regular classes at school has been proposed [13]. The current paper proposes a new method to extract objective measures from acceleration data. The method was applied to a “free living” environment in which participants performed their normal daily activities in their normal way without any extra requirement other than wearing two minimally invasive devices. These devices consisted of single tri-axial accelerometer which recorded acceleration signals from the wrist of the dominant arm and the ankle of the dominant leg of 11 children diagnosed with ADHD and 11 paired non-ADHD controls during a 24-h period. In the current paper we only analyze the periods when the children were at school, in order to maximize environmental consistency. Using a Convolutional Neural Network (CNN), we attempted to find acceleration patterns which distinguished ADHD children from typically developing non-ADHD controls. ADHD diagnosed children were further divided into a non-medicated group and a medicated group so that the effect of medication on movement could be isolated.

## 2. Related Work

Several research studies have already focused on the investigation of objective measures and methods that could be used in the assessment of ADHD patients based on body worn sensors. In previous studies, the analysis of the data gathered from several body worn sensors has been used both for hypothesis testing and for pattern recognition. Hypothesis testing based on wearable sensors data has shown statistically significant differences in several parameters measured from ADHD patients compared with non-ADHD controls [11,12]. Pattern recognition based on a predefined set of features calculated from an inertial measurement unit (IMU) showed good classification results in distinguishing ADHD patients from non-ADHD controls when performing a continuous performance test in a controlled environment [10].

The authors in [11] used objective measures to find statistically significant differences between children with ADHD and non-ADHD controls based on the forces applied during manipulation of a novel object and movement rhythmicity measured by sensors. The DSM-IV-TR criteria [6] was used to diagnose ADHD. A series of tests involving movements of the upper limb were administered while “off” medication. The results showed that significant differences could be observed between groups in movement rhythmicity, manipulation of a novel object, and dexterity. Moreover, the study showed that certain parameters such as the speed of execution of a requested movement and the measured tremor were unaffected.

The study in [12] also showed statistically significant differences between the way children with ADHD and typically developing non-ADHD controls move their upper limbs. In this study, a tri-axial accelerometer worn on the non-dominant arm was used to measure movements while the child attended three different school classes: art, language and mathematics. The authors characterized the intensity of the movements in the range of 0.5 to 2.8 g (g being the acceleration due to gravity) and showed that for the art class there were statistically significant differences in the regions of the low intensity movements (0.5–0.8 g) and the high intensity movements (1.3–2.8 g). However, in the language and mathematics classes, the authors observed smaller differences.

As previous studies found differences in the way ADHD children move compared to typically developing non-ADHD controls, O’Mahoni et al. attempted to develop an objective measure to help in the diagnosis of ADHD by characterizing the movements of each group in terms of a set of features, and then quantifying these features to classify each individual into likely ADHD and non-ADHD groups. The authors used two inertial measurement units (IMUs) comprising a tri-axial accelerometer and a gyroscope, one attached to the waist and the other to the ankle of the dominant leg, and took continuous measurements during an approximately 1-h visit to a psychiatric consultancy. The authors combined time domain as well as frequency domain calculations to extract 668 different features. Using a support vector machine (SVM), the results showed up to 95% for both accuracy and sensitivity.

In summary, previous studies have shown statistically significant differences in the way children with ADHD and typically developing non-ADHD control children move as captured by body worn sensors attached to different parts of the body. Both dominant and non-dominant limbs have been used to record acceleration data. Moreover, previous studies have shown that, based on the results of the acceleration measures taken while performing a predefined and controlled set of activities, typically developing non-ADHD controls and non-medicated children with ADHD can be automatically classified with a relatively high accuracy. In the current paper, we extend these studies by using two minimally invasive tri-axial accelerometers to record movement data in a “free-living” environment while at school. By “free-living”, in the context of this study, we mean that no additional requirements was given to participants. They followed their normal, everyday activities. Moreover, there were no other requirements for two participants from two different schools (or from the same school but monitored on different days) to follow (a similar script of activities was not required). Although a first study presented in this paper only focuses on the data of two accelerometers (worn at school on both dominant limbs), a complete dataset including 4 tri-axial accelerometers during an entire day period, one on each wrist and ankle has been recorded., We have only used the data from the dominant hand and leg in order to simplify the data measuring and handling process in this first study. Moreover, in order to compare results in a similar environment, only acceleration data while at school was analyzed. The complete dataset of all 4 tri-axial accelerometers for the full day will be presented in subsequent papers. Instead of predefining a set of features to be computed from the sensor data, a Convolutional Neural Network (CNN) was used to automatically learn the optimal features to separate children in each group. The use of CNNs in human activity recognition (HAR) applied to wearable sensor acceleration data has recently shown promising results compared to other non-deep learning approaches based on hand-crafted features [14,15,16,17,18]. Previous studies have generated CNN input images from windowed segments of acceleration data by using the time series information to generate the horizontal image axis and each tri-axial component of each accelerometer [14,18], or the combination of different sensors [15,16], or the combination of the accelerometer and gyroscope of a single IMU [17] to generate the associated vertical image information. In this paper we propose a different and novel approach to generate acceleration images that both produces square images and is able to compensate for sensor misplacements and movement drift by finding the acceleration projections over a geo-referenced space. The tri-axial acceleration is converted into the movement related linear acceleration by subtracting an estimation of the gravity acceleration. Using an estimation for the gravity force from the sensed acceleration data to compensate different sensor orientations and obtaining sensor orientation invariant information has been used in previous research studies such as [19,20,21] for human activity recognition. The estimated linear acceleration is then decomposed into its vertical (gravity acceleration aligned) and horizontal components. Using a window of 5 consecutive seconds of acceleration data, a squared acceleration image is generated with all the acceleration samples (using the vertical and horizontal acceleration values to select the pixel in the image to modify). Other approaches have been previously used in related literature to compute orientation invariant data from wearable tri-axial accelerometers such as [22] which uses the inner products of two acceleration samples to compute sensor orientation free features. Our proposal uses a similar approach but uses an estimation of the gravity acceleration vector in order to project acceleration samples onto a geo-referenced coordinate system. Acceleration images are therefore computed in a geo-referenced horizontal-vertical space. A different approach could have been, as described in [23], to use the autocorrelation matrix from the FFT transform to provide a rotation free measurement of three-dimensional measurements. The time dimension for the generation of the acceleration image would then be transformed into a frequency domain image but a geo-referenced time-based approach has been used in this paper in order to minimize the required computations. Computing acceleration images instead of using raw data series is also an important factor for the expected results. Previous research studies applying CNN to Human Activity Recognition (HAR) such as [14,15,16,17,18] has shown that a CNN is able to learn common acceleration patterns based on raw acceleration data. The results may be improved by transforming raw data into computed features. By using a simple architecture, the authors in [14] obtained even better results than previous papers based on more complex architectures but only using the raw acceleration data showing that the way in which the acceleration image is computed plays a very important role in the results that can be achieved. Finally, we have taken measures from non-medicated and medicated children with ADHD. While the CNN has been trained with children with ADHD who are unmedicated and typically developing non-ADHD controls, once trained, the CNN has been used to evaluate how close children with ADHD who are taking medication become to a typically developing non-ADHD control pattern. 

## 3. Materials and Methods

### 3.1. Participants and Demographics

#### 3.1.1. Inclusion Criteria

Participants were children and or young people aged 6–15, diagnosed with ADHD as reported by their parents who confirmed they had been fully assessed and diagnosed by the Paediatric Neurodisability or Child and Adolescent Menytal Health Services. Age matched controls were also aged 6–15, did not have a diagnosis of ADHD, and were not siblings of a child with diagnosed ADHD. (ADHD is highly heritable)

#### 3.1.2. Recruitment, Setting and Data Collection

Members of the research team recruited participants diagnosed with ADHD living in South Yorkshire via a community group for parents of children with ADHD. Parents were referred to these groups by their child’s clinician. A convenience sample of control participants was recruited using a snowball sampling method. 

All participants were visited in their homes by a member of the research team. The parent/legal guardian provided informed consent to confirm their child’s participation in the study. The children also signed assent forms to agree to participate. Additionally, the parents/legal guardians completed a SNAP IV questionnaire to indicate the current severity of their child’s hyperactive behaviors. The researcher placed a labelled sensor (embedded in a black sweat band) on each wrist and ankle of every child which were worn for 24 h including when they were in bed. Parents were shown how to position the sensors in the wristband should this be required i.e. if the sensors came out or the participant removed them to ensure they were in the correct position and orientation during the 24 h. Sensors were removed for bathing and showering. Parents were asked to record if the sensors had been removed and for what reason. This would then explain any data anomalies such as periods of complete inactivity.

#### 3.1.3. Demographics

A set of 22 children in 11 pairs were recruited in the first phase (more children will be recruited for future studies). Among the 11 children diagnosed with ADHD, five were not taking medication for their ADHD when data was recorded and six were on medication. Table 1 and Table 2 show the demographic data for the children participating in the first phase of the experiment. 

The mean short SNAP IV score [24] has been added to illustrate the severity of ADHD in each child. The pairing is shown in Table 3. All of the 22 children attended school the day in which data was recorded and performed the regular activities of a normal day at school. The recordings were conducted in the same season (October–November) 2017.

Participants with ADHD were recruited from a managing ADHD group in Sheffield. Referral to this group is only available to parents of children diagnosed with ADHD who have been assessed formally by the Paediatric Neurodisability or Child and Adolescent Mental Health Services. in Sheffield Childrens NHS Foundation Trust. This involves a standard assessment and diagnosis process which adheres to NICE guidelines and the DSM 5 criteria. Participants’ parents also completed a SNAP symptom questionnaire to assess their current symptoms. Naturally some control participants may be undiagnosed and may be near to the threshold of the SNAP questionnaire but this does not confirm a diagnosis of ADHD.

### 3.2. Ethics

Ethics approval was provided by the School of Health and Related Research (ScHARR) ethics committee at the University of Sheffield (Reference: 013209). Participant information sheets were written for different age groups to ensure all participants understood the study. 

### 3.3. Sensors and Data Gathering

**Figure 1:**
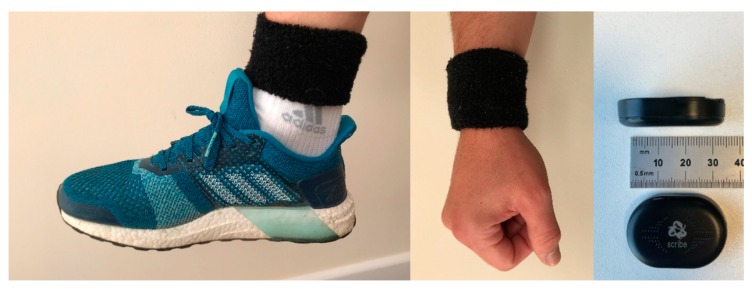
Positioning of the sensors.

The sensors used were two Runscribe™ inertial sensors (Scribe Labs, San Francisco, CA, USA) containing a tri-axial accelerometer which were applied to the wrist and ankle of the dominant arm and leg by aligning their long axis with limb and placing them within an elasticated ‘sweat’ band, see Figure 1. The sampling frequency for each sensor was set at 10 Hz with the addition of a low pass anti-aliasing filter at 5 Hz. The relatively low sampling frequency was chosen to allow 24 h collection of raw accelerometer data: this is expected to adequately characterize the movement patterns as the large majority of the power in voluntary human arm movement is below 2 Hz [25], although there may be low amplitude movements up to 8.4 Hz for occasional maximal speed movements [26]. Synchronization of sensors was through simultaneous initialization. The sensors’ measurement range was ±16 g, which was sufficient to avoid saturation (confirmed by inspection of the collected data). As previously stated, participants were asked to wear the sensor for a 24 h period (after which the sensor was programmed to stopped collecting). Parents were instructed to replace the sensors in the correct location and orientation if the child removed them at night. Adherence was confirmed by inspection of the accelerometer traces: if there was a discrepancy the collection was repeated. After the sensors were returned they were downloaded via a cable and the data analyzed. 

### 3.4. Data Processing

The data for each tri-axial accelerometer was used to compute 2D acceleration images based on a 5-s sliding window with 50% overlap. A Convolutional Neural Network (CNN) was trained based on the generated 2D acceleration images to automatically recognize common patterns in acceleration data that optimally separated non-medicated children with a diagnosis of ADHD from typically developing controls. In order to generate a 2D image out of each 5-s acceleration window, the movement related acceleration is projected over a geo-referenced coordinate system. This process requires estimating the direction of the gravity force from the recorded acceleration samples taken in the body anchored sensor coordinate system. A moving average based low pass filter is used to extract the gravity caused acceleration from the raw acceleration signal. The movement related acceleration is estimated by subtracting the gravity caused acceleration from the sensor raw data. The vertical movement related acceleration can then be estimated by calculating the dot product of the movement related acceleration and the unitary gravity vector. Once the vertical moment related acceleration has been estimated the horizontal (parallel to the ground) acceleration magnitude can be estimated by subtracting the vertical acceleration to the complete movement related acceleration. Once the vertical and horizontal acceleration components are computed, they can be projected into a 2D image. The details for the data processing process is described in the following set of steps:

1. The tri-axial acceleration is sampled at 10 Hz. Each sample therefore comprises 3 acceleration values (one per axis). The sample “*i*” can therefore be represented as $\vec{a_{t}\left(i\right)}=\left(a_{tx}\left(i\right),a_{ty}\left(i\right),a_{tz}\left(i\right)\right)$
2. The gravity force vector for the sample number “*i*” in the sensor coordinate axes is estimated by using a moving average based low pass filter using the following Equation (similar to the method proposed in [21]): $\vec{g{\left(i\right)}^{\prime }}=\frac{\sum_{j=i-\frac{N}{2}}^{j=i+\frac{N}{2}}\left(a_{tx}\left(j\right),a_{ty}\left(j\right),a_{tz}\left(j\right)\right)}{N+1}$, where *N* = 50 in our case so that a sampling rate of 10 Hz produces a 5 s data window as proposed in [14].
3. The acceleration caused by the movement of the limb is estimated according to: $\vec{a_{m}\left(i\right)}=\vec{a_{t}\left(i\right)}-\vec{g{\left(i\right)}^{\prime }}$
4. $\vec{a_{m}\left(i\right)}$ is then divided into vertical and horizontal acceleration components following the Equation: $\vec{a_{v}\left(i\right)}=\;\frac{\vec{a_{m}\left(i\right)}\cdot \;\vec{g{\left(i\right)}^{\prime }}}{{\vec{g{\left(i\right)}^{\prime }}}^{2}}\vec{g{\left(i\right)}^{\prime }}$ and $\vec{a_{h}\left(i\right)}=\vec{a_{m}\left(i\right)}-\vec{a_{v}\left(i\right)}$
5. A 5 s sliding window (51 samples for a sampling rate of 10 Hz) is used (similar to [14]) to generate an acceleration image using the following pseudo-code (for the time frame centered at sample number “*i*”):
  - Let img be a 28 by 28 image initialized to all zeros
  - for *j* = $i-\frac{N}{2}$ to $i+\frac{N}{2}$, *N* = 50
    - indexv = $floor\left(1+9\times min\left(3,\frac{\vec{a_{v}\left(j\right)}}{\vec{g{\left(j\right)}^{\prime }}\;}\right)\right)$
    - indexh = $floor\left(1+9\times min\left(3,\frac{\vec{a_{h}\left(j\right)}}{\vec{g{\left(j\right)}^{\prime }}\;}\right)\right)$ *
    - img(indexh,indexv) = 1
6. Store the acceleration image, move the window 2.5 s by updating the value $i=\;i+\frac{N}{2}$ and continue with step 5.
7. The acceleration images are computed for all participants while at school (from 9:00 to 15:00). Part of the data are used to train the CNN and part for validation as described in the following subsections

The resulting image is 28 by 28 and uses a geo-referenced coordinate system. The size of 28 by 28 for square images feeding a CNN has been widely used in previous research studies for image recognition and in benchmark image databases such as the MNIST database of handwritten digits [27]. The acceleration images are generated using the estimated vertical and horizontal accelerations trying to minimize the effect of the different placements of the sensors for each participant and the relative to the body sensor moves caused by a loose sensor strap. The computed acceleration images were used both for hypothesis testing and for automatic classification of new data as described in the following subsections.

#### 3.4.1. Hypothesis Testing

**Figure 2:**
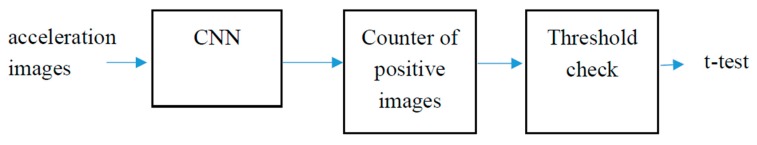
Hypothesis testing schema.

As described earlier in this paper, hypothesis testing based on wearable sensor data has shown statistically significant differences in several parameter measures from ADHD patients as compared with typically developing non-ADHD controls [11,12]. Previous studies have focused on controlled environments in which the participants were told to execute a set of activities in a predefined order in a controlled setting. In this paper, we want to investigate if there are differences in the movement patterns between non-medicated children with ADHD and paired typically developing non-ADHD controls while in a “free living” environment during 6 h at school, using movement data from the wrist and ankle of the dominant limbs. For each participant with ADHD a similar typically developing non-ADHD control was selected. The null hypothesis was that there were no differences in the way children with ADHD and typically developing non-ADHD controls moved, computed using the acceleration images described in the previous section. The procedure is illustrated in Figure 2.

We used the recommendations provided in [14] in order to design the CNN. We used 3 convolutional layers and a final fully connected layer. The CNN has been configured in Matlab [28] with the following parameters:

layers = [

  imageInputLayer([28 28 1])

  convolution2dLayer(3,8,‘Padding’,1)

  batchNormalizationLayer

  reluLayer

  maxPooling2dLayer(2,‘Stride’,2)

  convolution2dLayer(3,16,‘Padding’,1)

  batchNormalizationLayer

  reluLayer

  maxPooling2dLayer(2,‘Stride’,2)

  convolution2dLayer(3,32,‘Padding’,1)

  batchNormalizationLayer

  reluLayer

  fullyConnectedLayer(2)

  softmaxLayer

**Figure 3:**
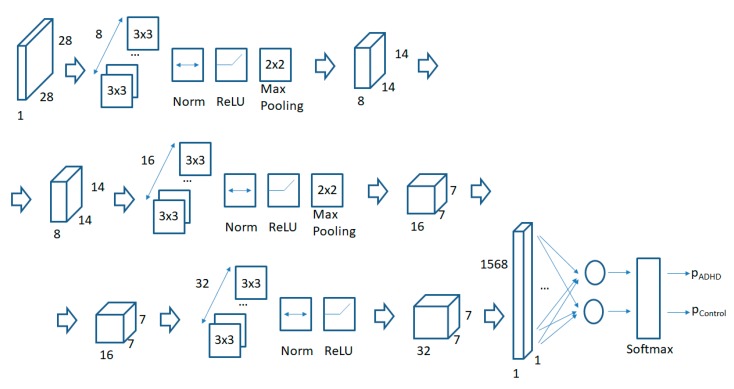
CNN architecture.

  classificationLayer];

The graphical representation for the architecture for the CNN is captured in Figure 3. The size of 28 by 28 for square images feeding a CNN has been widely used in previous research studies for image recognition and in benchmark image databases such as the MNIST database of handwritten digits [27]. All filters in the CNN are 3 by 3 by k matrices (being k the number of channels in the input images to the convolutional layer). A padding of “ceros” of size 1 is added to all edges in the output image so that the convolutional operation does not reduce the size of the vertical and horizontal dimensions by 2 units. A normalization operation is added after each convolutional layer and before the non-linear ReLU layer to improve the convergence in the training of the CNN. A final max polling layer is used at the end of the first 2 convolutional layers to reduce the vertical and horizontal sizes for the output images by a factor of 2. The number of channels is increased by a factor of 2 in each consecutive convolutional layer as the sizes of the horizontal and vertical dimensions are reduced by a similar factor of 2 as previously said. A final fully connected layer and a softmax layer assign a probability for each input image to one of the classes (ADHD or non-ADHD control). The default parameters have been used for the weight and bias learning rates in the training of the CNN.

The output layer assigns probabilities for each image to each group (ADHD or typically developing child) based on the softmax layer and selects the group with a higher probability. For the hypothesis testing algorithm, each acceleration image has been assigned to one of the groups 

The “leave one out” schema was used to train the CNN. The data from all children with ADHD and typically developing non-ADHD controls, except for one child, were used to train the CNN, with the left-out child used to validate the results. The CNN automatically learned common acceleration traces or patterns (represented as acceleration images) associated with children with ADHD and typically developing non-ADHD controls. For the child not used in the training phase, the trained CNN was used to classify each acceleration image into an ADHD or control group. As there were many acceleration images common to both groups, some of the images were miss-classified. A counter was therefore added after the CNN to count the number of positive images (acceleration images classified in the ADHD group) in a set of consecutive images. We have selected *n* = 20 as the size of the set of consecutive images to be computed together (52.5 s of data). A threshold *th (*n≥th≥1*)* is then used to assess whether or not a particular child belonged to the ADHD or the typically developing non-ADHD control group. Let *m* be number of acceleration images classified as ADHD in the set of *n* consecutive images. We determined if the particular child belonged to the ADHD group if m≥th.

The complete set of acceleration images for each pair of participants was used to label each 20 consecutive images as ADHD or control for each th=1:20. The bigger the threshold the lower the number of image segments marked as ADHD both children with ADHD and for typically developing non-ADHD controls. Each participant therefore received a value for each threshold and these values per threshold were used to calculate the *p* value of a 2-sample *t*-test. The null hypothesis was that there was no statistically significant difference in the values of segments marked as ADHD candidates.

#### 3.4.2. Automatic Classification Using CNNs

If statistically significant differences in the acceleration patterns of those with ADHD and typically developing non-ADHD controls were found, algorithms could be designed to apply pattern recognition to the data in order to classify unknown samples. As mentioned in the related work section, some machine learning algorithms based on a predefined hand-crafted set of features calculated from an inertial measurement unit (IMU) have already shown good classification results in order to automatically distinguish ADHD patients from typically developing non-ADHD controls when performing a continuous performance test (CPT) in a controlled environment [10] but there is a need to have similar methods that work well for data gathered in a “free living” environment. A novel method is proposed in this sub-section based on the data obtained from participants during their school hours. 

**Figure 4:**
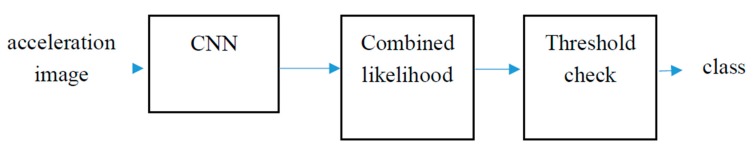
Classification schema.

The acceleration images previously described were used as input to a Convolutional Neural Network (CNN) as in the hypothesis testing scenario but the classification algorithm was modified in order to add the confidence of the CNN while classifying a new acceleration image to the decision process. The proposed schema is shown in Figure 4.

The same CNN structure as shown in the previous subsection has been used (as captured in Figure 3). The output layer of the CNN is a softmax layer which assigns probabilities to the output of the fully connected layer per class (logits) according to the following equation:

(1) $$\;p\left(i\right)=\frac{e^{z_{i}}}{\sum_{j}e^{z_{j}}}\;$$

where *i* represents the class (ADHD child or typically developing non-ADHD control) and $z_{i}$ are the outputs of the fully connected layer.

Both a 4-fold cross-validation and a leave-one-out schemas were used to validate results. In the 4-fold cross-validation approach, for each participant, 1.5 h of acceleration data were separated for validation purposes while 4.5 h were used in order to train the CNN. Both training and validation samples were selected in consecutive periods of time. For each image in the validation data, the likelihood of belonging to each class was calculated as shown in the previous Equation. The combined likelihood box sums the probabilities for all the images in the validation set for each participant according to the following Equation:

(2) $$\;cl\left(i\right)=\underset{j}{\sum }p{\left(j\right)}_{c=ADHD}-\underset{j}{\sum }p{\left(j\right)}_{c=Control}\;$$

where, in this case, *cl* is the combined likelihood, *i* is the participant number, *j* iterates through all the images in the validation set for participant *i* and c represents the class (ADHD or control). In this case:

(3) $$\;p{\left(j\right)}_{c=ADHD}=1-p{\left(j\right)}_{c=Control}\;$$

The output of the combined likelihood box should be positive for true positives (participants with ADHD classified as such) and negative for true negatives (typically developing non-ADHD controls classified as such). In this case, the threshold will be zero (*th* = 0) and the further positive or negative the output is, the greater the confidence in the result of the classification process.

The major limitation in the 4-fold cross-validation schema is that the CNN sees data from all participants in the training phase so that particular patterns from each individual are learned and, therefore, the predictions of the trained CNN will be good if the acceleration patterns for each user are repeated over time. In order to validate the generalization of results for new users, a leave-one-out validation schema has also been implemented following the same architecture as shown in Figure 4. In this case, the data (acceleration images) from all participants except one is used to train the CNN and the data from the left-aside participant is the used to validate results. The process is repeated for each participant.

## 4. Results

This section shows the results of applying the proposed data processing methods to the data gathered according to the information provided in the previous section. The hypothesis testing results are presented first, and the classification results follow.

### 4.1. Hypothesis Testing Results

In order to validate if there was a statistically significant difference between the acceleration images captured as described in Section 3 from typically developing non-ADHD controls and from participants with ADHD, a leave one out approach has been followed in order to train the CNN in Figure 2. The number of images classified as belonging to the ADHD group in a frame of 20 consecutive images was then compared with a threshold. The entire frame is marked as positive if the number of ADHD classified acceleration images is greater or equal to that threshold. The 20 images frame is the moved 20 images to cover the next set of acceleration images and the process is iterated for the 6-h period from 9:00 to 15:00 h (while children/young people were in school). The process is repeated for each participant and for each threshold and a final *t*-test (as described in Figure 2) is carried out. The 2-sample *t*-test has been applied to the data from all participants with ADHD (experiment group) versus the data from all typically developing children (control group) and to the data of only non-medicated participants (experiment group) versus the data from all typically developing children (trying to isolate the effect that medication has on the way individuals with ADHD move). The results for the *p*-values for each threshold are presented in Table 4. When the data of both medicated and non-medicated children with ADHD is taken into account, there is only statistical significance in results for the wrist acceleration images. The *p*-values for the wrist sensor in the case of all participants are captured in the first results column in Table 4. The null hypothesis could be rejected for a value of α=0.05 (the null hypothesis being that there is no difference in the mean values for both ADHD and control children) for all values of *th* from 5 to 19. The results for the ankle acceleration images are not captured in Table 4 since they do not show a statistical significance for any value of *th*. The last two columns in Table 4 capture the *p*-values in the case that only non-medicated children are taken into account for the ADHD group. Appropriate treatment with medication for ADHD is likely to affect movements and may therefore introduce some distortion in the results. The last two columns in Table 4 show that there is a statistical significance in the *p*-values for both the wrist and ankle acceleration images for all values of *th* for a value of α=0.05.

Table 4 captures the intuitive result that if the value of the threshold *th* is low, there will be more segments for a typically developing non-ADHD control participant that will be classified as ADHD and therefore the value of the computed *p*-value will be non-optimal. On the other hand, if the value of the threshold tends to the size of the entire set of consecutive images, there will be many children with ADHD that will be considered as belonging to the control group leading again to a non-optimal number for the computed *p*-value. 

The results in [12], using a tri-axial accelerometer to monitor the movement differences between children with ADHD and typically developing children in arts, language and math courses, using a similar *t*-test, only found statistical significance for the art course, but the authors only used the mean ratios of activity acceleration in a 1-min epoch between the groups, which differed significantly (*p* < 0.05) at the 0.5–0.8 G and 1.3–2.8 G regions (G being the gravity caused acceleration). Authors in [11] also found statistical significance for the differences in which children with ADHD lifted a novel object using a lower grip force (*p* = 0.036). The authors in [11] also found statistical significance in the way both groups held a novel object with a more variable grip force (*p* = 0.003). Other parameters also showed statistically significant differences such as rhythmicity of finger tapping (*p* = 0.008), dexterity (*p* = 0.007) and aiming and catching (*p* = 0.042) movements. The results in our study extend these previous studies to a different setting in a familiar environment which minimizes the requirements for participants.

### 4.2. Classification Results

The *p*-values from the previous section indicate that there are statistically significant differences in the acceleration data from both groups. In this section, we present the results of using the schema proposed in Figure 4 in order to automatically classify children into their group.

#### 4.2.1. Classification Results Using a 4-Fold Cross-Validation Schema

In this first case of using a 4-fold cross-validation schema, the CNN was trained with 4.5 h of acceleration data from each of the ADHD non-medicated participants and the same amount of data from the typically developing non-ADHD control group. A distinct 1.5 h of acceleration data for the same children were used for validation. All children wore accelerometers during the entire period at school and were instructed to follow the normal day at school. Each acceleration image in the validation set was fed into the CNN and the probability assigned after the softmax output layer for each group (ADHD, as learned from non-medicated data, or typically developing control) was recorded per image. A final score per participant was calculated by adding the probabilities for all the images per group so that the bigger the difference the more likely it is that this particular child belongs to the most likely class (as shown by Equation (2). Table 5 shows the classification results for all non-medicated children with ADHD and all typically developing non-ADHD controls following the proposed method for the wrist acceleration data. All participants were correctly assigned into their group except for one of the controls who was assigned to the ADHD group (accuracy = 0.9375, sensitivity = 1, specificity = 0.9091).

Similar results for the data obtained from the ankle accelerometer are presented on Table 6. In this case, there is again only one miss-classified participant, but in this case one of the ADHD non-medicated participants was assigned to the typically developing non-ADHD control group (accuracy = 0.9375, sensitivity = 0.8, specificity = 1).

The proposed automatic classification schema could also be used to measure the effect that medication has on ADHD children. i.e., does medicine move the acceleration patterns of ADHD children towards similar patterns to typically developing non-ADHD controls? Table 7 and Table 8 show the results of applying the classifier trained with the control and non-medicated ADHD children to medicated ADHD participants. For the wrist accelerometer we see that the patterns of 4 out of the 6 medicated participants are classified as more likely to be from a typically developing non-ADHD control than from a non-medicated ADHD child. The results for the ankle accelerometer show that only two medicated children are classified as typically developing non-ADHD controls. Moreover, the child which is correctly classified in both cases is the one with a higher SNAP score (having the maximum value of 3) and the child which is wrongly classified in both cases is the one with the smaller SNAP score, close to a non ADHD child (a score of 1.66 on the scale from 0 to 3 from which a child is diagnosed as ADHD, where a score of 1.44 or above is indicative that the child may be suffering ADHD).

#### 4.2.2. Classification Results Using a Leave-One-Out Validation Schema

In the leave-one-out validation, we used all the acceleration images for all the typically developing non-ADHD controls and all the non-medicated ADHD diagnosed children while in school except for one of them at a time in order to train the CNN. The acceleration images for the left aside child were used for validation. The process was repeated for each child.

Table 9 shows the results for the wrist acceleration images and Table 10 captures the results for the ankle data. In both cases, all controls are correctly classified. In the case of the acceleration images based on the wrist accelerometers from the dominant hand, ADHD participants 1 and 12 and misclassified. The results for the ankle show that only ADHD participant 12 is misclassified. 

Table 9 and Table 10 show that there are generalizable patterns that are common to the majority of ADHD diagnosed non-medicated participants that can be learned as opposed to generalizable patterns from typically developing children. The architecture proposed in this manuscript based on using a CNN over georeferenced square acceleration images is able to automatically classify a child not used in the training of the algorithm with an accuracy of 0.875 (sensitivity = 0.6, specificity = 1) for the wrist sensor and an accuracy of 0.9375 (sensitivity = 0.8, specificity = 1) for the ankle sensor. The results are compared with those in related previous studies in Table 11. Our approach is able to outperform previous experiments without requiring the implementation of a monitored and pre-defined set of activities but monitoring children while at school performing different (not pre-defined) activities is a free way (children were not specified to follow any particular requirement).

## 5. Discussion

These results expand those reported by previous papers using sensors to measure differences between children diagnosed with ADHD and paired typically developing non-ADHD controls to a “free living” environment when participants were following their normal activities in their own school, A novel method that uses acceleration data in both dominant limbs (separately, to avoid synchronization issues and clock sampling drifts between the two sensor devices) to feed a deep learning architecture based on a Convolutional Neural Network (CNN) has shown both that there are statistically significant differences in the way non-medicated children with ADHD move while in school compared to paired typically developing non-ADHD controls, and that these differences can be used to automatically classify children into the correct group with an accuracy of 0.9375 using a leave-one-out approach to assess the generalization of results. This method could be used as a complementary tool in the diagnosis of the disorder and to perform a non-intrusive follow up monitoring e.g. during medication optimization.

The acceleration patterns capture all the different movements performed by each child. Using a 5 s window has been shown to be able to capture the details in sporadic movements as well as movements with some periodicity. Other window sizes could be explored in future work in order to find other movement patterns.

Previous studies using acceleration data to characterize the movement patterns of ADHD children have shown differences both when using the data from the dominant limbs as well as the non-dominant limbs. While fine and precision movements tend to be executed using the dominant limbs (with a secondary support of the non-dominant limbs in some cases), involuntary or background patterns are more easily observed from the non-dominant limbs since the acceleration data in non-dominant limbs tends to have less influence from the execution of precision movements. In this paper, we have looked for characterizing patterns in both dominant arm and leg. The results corroborate that there are statistically significant differences that can be used to differentiate between children with ADHD and typically developing non-ADHD controls including the information in many tasks that are executed mainly with the dominant hand while in school (such as writing). The data from the non-dominant limbs have also been recorded and will be used to complement results in future studies.

Many of the acceleration images show scores at the output of the Convolutional Neural Network (CNN) which assign similar probabilities to both classes. This means, that many of the acceleration patterns are similar for all the children. However, there are some acceleration images which are more characteristic of one group or the other. A further study of these characteristic acceleration segments could provide a further insight to assess the ways in which ADHD affects movement.

The results also show that medication use modifies acceleration patterns in children with ADHD making their acceleration patterns more like those found in normal children. If the SNAP IV score indicates a high severity in the disorder, the acceleration patterns tend to retain more ADHD like characteristics. However, for ADHD children with smaller SNAP score values, medication can influence the way they move to be closer to typically developing non-ADHD controls. This may indicate levels of optimization of medication and could be useful clinically.

The study has shown important insights in a group of 22 children during their school hours. The study will be extended to more children for their entire day in future work to further validate the generalization of results. The study will also be extended to deal with other limitations of the current results by increasing the recording time to several days for each participant, and increasing the sampling frequency to 50 Hz, to monitor the adherence to wear the sensor and to assess the effects of medication over time. 

## Figures and Tables

**Table 1:** Participant demographics for ADHD diagnosed participants.

ID	Age	Gender	On Medication	Mean SNAP Score ^1^
ADHD 1	12	Male	No	1.33
ADHD 3	12	Male	No	2.22
ADHD 4	10	Male	No	2.44
ADHD 5	12	Male	Yes	1.77
ADHD 6	7	Male	Yes	3
ADHD 9	7	Male	Yes	1.88
ADHD 10	9	Male	No	2.66
ADHD 11	7	Female	Yes	2.11
ADHD 12	7	Male	No	2.44
ADHD 14	12	Male	Yes	1.66
ADHD 15	12	Male	Yes	2

^1^ SNAP score [24]. The value 0 is the lowest score that can be obtained on the SNAP questionnaire and 3 is the highest score. A score of 1.44 or above supports a diagnosis of ADHD.

**Table 2:** Participant demographics for paired control participants.

ID	Age	Gender	SNAP Score ^1^
Control 1	12	Male	0.44
Control 2	10	Male	0.77
Control 5	7	Male	0.22
Control 6	9	Male	0.88
Control 7	12	Male	0.22
Control 8	11	Male	0
Control 11	10	Male	1.55
Control 13	7	Male	0.22
Control 14	6	Female	0.55
Control 15	9	Male	0.88
Control 16	10	Male	1

^1^ SNAP score [24].

**Table 3:** Participant pairing.

ID ADHD Participant	ID Control Participant
ADHD 1	Control 1
ADHD 3	Control 11
ADHD 4	Control 2
ADHD 5	Control 7
ADHD 6	Control 5
ADHD 9	Control 13
ADHD 10	Control 6
ADHD 11	Control 14
ADHD 12	Control 15
ADHD 14	Control 16
ADHD 15	Control 8

**Table 4:** *p*-values for different threshold values.

Th	*p*-Value Wrist Accelerometer All Data	*p*-Value Wrist Accelerometer Non-Medicated Only	*p*-Value Ankle Accelerometer Non-Medicated Only
1	0.083882	0.000398	0.002628
2	0.079884	0.000179	0.003048
3	0.061272	0.000114	0.002297
4	0.050620	0.000085	0.001944
5	0.045805	0.000078	0.001664
6	0.041795	0.000073	0.001400
7	0.037090	0.000076	0.001129
8	0.033110	0.000073	0.000849
9	0.029275	0.000077	0.000675
10	0.027535	0.000098	0.000565
11	0.026288	0.000118	0.000498
12	0.025612	0.000171	0.000452
13	0.025405	0.000288	0.000433
14	0.024332	0.000578	0.000434
15	0.024096	0.001293	0.000459
16	0.025293	0.003382	0.000557
17	0.026486	0.007844	0.000696
18	0.031052	0.015559	0.000817
19	0.041961	0.025984	0.000629
20	0.065378	0.035712	0.000340

**Table 5:** 4-fold cross-validation results based on the wrist acceleration data (non-medicated only).

Participant ID	Classified as
ADHD1	ADHD
ADHD3	ADHD
ADHD4	ADHD
ADHD10	ADHD
ADHD12	ADHD
Control1	Control
Control11	Control
Control2	Control
Control7	Control
Control5	Control
Control13	**ADHD**
Control6	Control
Control14	Control
Control15	Control
Control16	Control
Control8	Control

**Table 6:** 4-fold cross-validation results based on the ankle acceleration data (non-medicated only).

Participant ID	Classified as
ADHD1	ADHD
ADHD3	**Control**
ADHD4	ADHD
ADHD10	ADHD
ADHD12	ADHD
Control1	Control
Control11	Control
Control2	Control
Control7	Control
Control5	Control
Control13	Control
Control6	Control
Control14	Control
Control15	Control
Control16	Control
Control8	Control

**Table 7:** 4-fold cross-validation results based on the wrist acceleration data for medicated ADHD children.

Participant ID	SNAP Score	Classified as
ADHD5	1.77	Control
ADHD6	3	ADHD
ADHD9	1.88	ADHD
ADHD11	2.11	Control
ADHD14	1.66	Control
ADHD15	2	Control

**Table 8:** 4-fold cross-validation results based on the ankle acceleration data for medicated ADHD children.

Participant ID	SNAP Score	Classified as
ADHD5	1.77	ADHD
ADHD6	3	ADHD
ADHD9	1.88	Control
ADHD11	2.11	ADHD
ADHD14	1.66	Control
ADHD15	2	ADHD

**Table 9:** Leave one out validation results based on the wrist acceleration data (non-medicated only).

Participant ID	Classified as
ADHD1	**Control**
ADHD3	ADHD
ADHD4	ADHD
ADHD10	ADHD
ADHD12	**Control**
Control1	Control
Control11	Control
Control2	Control
Control7	Control
Control5	Control
Control13	Control
Control6	Control
Control14	Control
Control15	Control
Control16	Control
Control8	Control

**Table 10:** Leave one out validation results based on the ankle acceleration data (non-medicated only).

Participant ID	Classified as
ADHD1	ADHD
ADHD3	ADHD
ADHD4	ADHD
ADHD10	ADHD
ADHD12	**Control**
Control1	Control
Control11	Control
Control2	Control
Control7	Control
Control5	Control
Control13	Control
Control6	Control
Control14	Control
Control15	Control
Control16	Control
Control8	Control

**Table 11:** Accuracy comparison with previous results in controlled settings.

Reference	Sensors Used	Description of the Experiment	Accuracy
[9]	Electrodes located in the midline of the head	Controlled experiment comprising audiovisual sitimuli	Between 73.91% and 91.30%
[10]	A combination of two IMUs (comprising a tri-axial accelerometer and a gyroscope), and the age, gender and the result of the test of variables of attention (T.O.V.A.) test (http://www.tovatest.com/)	Data was recorded during the visit to a psychiatric consultancy performing a pre-defined set of activities	Between 83.72% and 95.12%
Our research	Two tri-axial accelerometers	Children attending activities at school, including breaks and meals (including children in different schools and different types of activities in different days of the week)	Between 87.5% and 93.75%
